# Does Internet Use Impact the Health Status of Middle-Aged and Older Populations? Evidence from China Health and Retirement Longitudinal Study (CHARLS)

**DOI:** 10.3390/ijerph19063619

**Published:** 2022-03-18

**Authors:** Liqing Li, Haifeng Ding, Zihan Li

**Affiliations:** 1 School of Administration and Law, Hunan Agricultural University, Changsha 410100, China; liliqing1136@163.com; 2 School of Economics and Management, Zhejiang Ocean University, Zhoushan 316000, China; ZH09537@163.com

**Keywords:** internet use, middle-aged and older population, self-assessed health, chronic disease status, China

## Abstract

In the context of both rapid technological development and increasing aging, the relationship between technological development and the health of the middle-aged and older population is gradually receiving academic attention. This study empirically examined the health consequences of the Internet for the middle-aged and older population in China using data from the 2018 China Health and Retirement Longitudinal Study. The results indicated that Internet use was effective in improving the self-assessed health and chronic disease status of the middle-aged and older population. However, the effect of Internet use on the improvement of chronic disease conditions in this population was more pronounced than self-assessed health. In the heterogeneity analysis, the effect of Internet use on the health of female and middle-aged adults was more significant than that of male and older adults aged >60 years. This paper also used a propensity score matching model to eliminate the endogeneity problem caused by sample selectivity bias. The results revealed that the propensity score matching model analysis was more robust. Moreover, if sample selectivity bias was not eliminated, the effect of Internet use on the improvement of self-assessed health in the middle-aged and older population would be underestimated, whereas the effect of Internet use on the chronic disease status of the middle-aged and older adults would be overestimated.

## 1. Introduction

Health is a basic condition for economic and social development [1]. Recently, with the continuous development of China’s social, people’s living standards continue to improve, and the health status of residents has greatly improved [2,3]. However, because of material conditions, chronic diseases such as hypertension, stroke, and other cardiovascular diseases have seriously affected the health of Chinese residents [4,5]. With the gradual degradation of the body’s functions, the middle-aged and older population are much more likely to experience chronic diseases, accounting for the largest number of chronic diseases in China [6]. To this end, the Communist Party of China and the State Council have introduced policies aimed at improving the overall health of the population. In 2017, President Xi Jinping proposed the Health China strategy, which clearly stated that the national health policy should be improved to provide people with all-round and whole-cycle health services [7]. In 2020, the Fifth Plenary Session of the 19th Party Congress re-emphasized the comprehensive promotion of the Health China strategy [8]. The above series of policies profoundly indicates that the health of the population will be a key concern for China for a long period in the future.

Moreover, the rapid development of the Internet has brought new ideas and ways to improve the health of residents [9,10]. Internet technology has solved the limitation of time and space, as people can visit health-themed websites and applications anytime and anywhere to browse and obtain health information [11]. According to the statistical bulletin data on national economic and social development in 2020 released by the China Statistics Bureau [12], there are a whopping 989 million internet users in China. The number of people online ranked first in the world. In China, the middle-aged and older population often use Internet platforms for social interaction and entertainment. Through daily observation, many older people increase the opportunity to meet their children through WeChat and use short video platforms such as Jitterbug for daily entertainment, which greatly enriches the spare time and spirituality of the middle-aged and older population [13]. However, whether Internet use affects the health of the middle-aged and older population needs further verification from the perspective of empirical studies. Therefore, this paper will focus on the following questions: (1) Does Internet use affect the health of middle-aged and older population? (2) If so, is the effect positive or negative? (3) Are there differences in the effects of Internet use on the health of the middle-aged and older population across sexes and age groups?

The rest of the paper is structured as follows. The second part presents a review of the literature on the effect of Internet use on population health. The third part focuses on the materials and methods, including the introduction of data sources, design of key variables, and analysis strategies. The last part is the discussion and conclusion.

## 2. Literature Review

Two main perspectives in existing studies focus on the effects of Internet use on population health. First, Internet use can improve the population’s health. Li found that Internet use demonstrated significant positive effects on the health of rural adults through a survey of 7528 rural residents aged >16 years in China. They also found that adults improved their health by increasing social interaction and staying physically active through the Internet [14]. Lyu studied 7193 older adults aged 60–95 years and found that Internet use was positively associated with the self-assessed health of older adults, while social capital was an important mediator of this relationship [15]. According to Han, Internet use can improve the health of residents in multiple dimensions based on data from the 2017 China General Social Survey [16]. Yang and Ho reported that Internet use had a significant positive effect on residents’ health. Compared with not using the Internet, residents using the Internet were 3.9% more likely to rate themselves as very healthy, 2.7% more likely to be free of physical pain, 0.75% more likely to have a better mood, and 1.3% and 1.1% less likely to experience injury and hospitalization, respectively [17]. Similarly, Hunsaker found that frequent health-related Internet use may promote, improve, or maintain the health of young Americans [18]. Studies have also focused on the mental health of the population. For example, the Duplaga study revealed that Internet use was effective in improving adults’ well-being, promoting their mental health, and increasing more beneficial health behaviors [19]. Fu reported a more significant correlation between social media use and mental health than physical health in older adults [20]. Zhang found that Internet use significantly reduced depression levels and discontinued Internet use was not significantly associated with improvements in depression or life satisfaction [21]. In addition, Zhang [22], Zhao, Liu [23], and Wang [24] confirmed that Internet use is effective in improving the mental health of residents.

Second, Internet use is detrimental to health. In a study of 327 medical students in Turkey, Güzel found that increased Internet use can lead to various physical health problems, which can cause permanent damage to physical health [25]. Kojima found that Internet use was strongly associated with reduced time for sleeping, studying, and exercising and increased the risk of depression among Japanese adolescents [26]. The study by Kokka et al. revealed that problematic internet use has a negative impact on the sleep and health of teenagers in Athens [27]. Using 1431 respondents in the UK, Mars’ study found that excessive internet use increased the risk of depression in UK women (highest tertile vs lowest tertile OR = 1.41, 95% CI 0.90 to 2.20) [28]. Xie found that Internet use affected the mental health of older adults and increased their incidence of depressive symptoms [29]. The effect of Internet use was greater for women, young and middle-aged people, individuals with higher income, non-rural households, less-educated individuals, and those living with others. In a study of 1954 rural Chinese adolescents, Ning et al. found that increased time spent using the Internet significantly increased the rate of sleep deprivation and obesity among rural adolescents, thereby affecting their physical health [30].

In summary, we found that despite studies about the effects of Internet use on population health, no unified conclusion has been established thus far. Regarding research participants, studies have mostly focused on adults, older individuals, rural residents, and adolescents, while relatively few studies have focused on the middle-aged and older population. However, with the increasing trend of population aging in China, exploring the relationship between scientific and technological progress and the middle-aged and older population has great practical value to improve the health of the whole population and successfully realize the strategy of a healthy China. Therefore, this study explores the impact of Internet use on the health of middle-aged and older populations using the results of the latest China Health and Retirement Longitudinal Study (CHARLS). This provides evidence to further understand the relationship between scientific and technological development and population health and reference for government departments to develop population health-promotion strategies.

## 3. Materials and Methods

### 3.1. Data Sources

Data used in this study were derived from the 2018 China Health and Retirement Longitudinal Study (CHARLS). The survey was hosted by the National Development Research Institute of Peking University and co-organized by the China Social Science Research Center of Peking University. This database is one of the most commonly used databases in China to study the health of the middle-aged and older population and provides high-quality microdata representing households and individuals aged ≥45 years in China. The CHARLS survey began in 2011, and the survey randomly selected 17,000 respondents from 150 counties (cities and districts) and 450 villages (neighborhood committees) in 28 provinces (municipalities and districts) and approximately 10,000 households nationwide. According to the purpose of the study, we selected people aged ≥45 years as the analysis sample. Through the process of removing missing values, processing abnormal values, and data screening, we finally obtained 10,778 valid samples.

### 3.2. Variable Design

#### 3.2.1. Dependent Variable

In this study, the dependent variable was the health of the middle-aged and older population. With reference to previous studies [31,32,33], we selected self-assessed health and chronic disease status to jointly measure the health of the middle-aged and older population. Self-assessed health is a subjective judgment of an individual’s overall health status within a short period, and several studies have confirmed the representativeness and scientific validity of self-assessed health. Unlike self-assessed health, chronic disease status is an objective measure of the health of the middle-aged and older population and is more reflective of the current and objective levels of health. For self-assessed health, in the 2018 CHARLS database, the responses to the question ‘How do you think your health is?’ were categorized as very good, good, general, bad, and very bad. In this study, very bad was assigned as 1, bad as 2, general as 3, good as 4, and very good as 5, and the increasing value of 1–5 indicates the higher health levels of the middle-aged and older population. Chronic disease status was measured by the question ‘Do you have a chronic disease?’ A total of 14 chronic diseases were included, namely, hypertension, dyslipidemia, diabetes, malignancy, lung disease, liver disease, heart disease, stroke, digestive disease, mental illness, Alzheimer’s disease, arthritis, stomach disease, and asthma. If the respondent has any one of these diseases, he or she is chronically ill and is assigned a value of 1, otherwise 0.

#### 3.2.2. Independent Variables

In the 2018 CHARLS database, the independent variable was assessed using the question ‘Which of the following tools do you use to access the Internet?’ These tools included desktop computers, laptops, tablets, cell phones, etc. If the respondent chooses any one of these tools, a value of 1 was assigned to their use of the Internet; otherwise, a value of 0 was assigned. To perform robustness tests, we also determined whether Internet use for social activities is a proxy variable for robustness testing. This aspect was evaluated by the question, ‘Have you done any of the following social activities in the past month? The question included 10 response options; a value of 1 was assigned if the respondent chose the Internet option, indicating that he or she uses the Internet; otherwise, it was 0.

#### 3.2.3. Control Variables

Grossman’s health needs model [34] suggests that factors affecting health are mainly socioeconomic, behavioral, and environmental, including medical services, education level, age, sex, marital status, and personal behavior (e.g., smoking and drinking). Therefore, we referred to Grossman’s health needs model and combined it with related studies [35,36] to include factors, such as sex and age of the middle-aged and older population, as control variables. According to the descriptive statistics, the mean age of the sample was 63.723 years. Regarding Internet use, only 13.45% of the middle-aged and older population were using the Internet, which indicated the big gap with China’s Internet penetration rate (57.7%). Regarding marital status, the highest proportion of the participants was married, with 85.04%. In terms of education level, elementary school education had the highest percentage of 41.48%, followed by people with secondary school education, accounting for 27.26%, and illiterate people with 26.72%. The specific descriptive statistical results are presented in Table 1.

### 3.3. Analysis Strategies

Since the explanatory variables are category variables, different econometric models will be set up to analyze the health consequences of the Internet for the middle-aged and older population. For the five-category variables, the following OProbit regression model [37] was set up:

(1) $${\mathrm{Health}}_{i}^{*}=\gamma +\beta {\mathrm{Internet}}_{i}+{\mu \theta }_{i}+\varepsilon_{i}$$

(2) $${\mathrm{Health}}_{i}=\{\begin{array}{l} 1,\;{\mathrm{Health}}_{i}^{*}\;\leq \;C1\;\\ 2,\;C1\;<\;{\mathrm{Health}}_{i}^{*}\;\leq \;C2\\ 3,\;C2\;<\;{\mathrm{Health}}_{i}^{*}\;\leq \;C3\\ 4,\;C3\;<\;{\mathrm{Health}}_{i}^{*}\;\leq \;C4\\ 5,\;C4\;<\;{\mathrm{Health}}_{i}^{*} \end{array}$$

In Equation (1), ${\mathrm{Health}}_{i}^{*}$ is a latent variable for the health level of middle-aged and older adults, ${\mathrm{Internet}}_{i}$ indicates Internet use by middle-aged and older adults, $\theta_{i}$ is a control variable that affects the health status of middle-aged and older adults and $\varepsilon_{i}$ is a random disturbance term. γ is the constant term, β is the regression coefficient and μ is the coefficient of the control variable. In Equation (2), ${\mathrm{Health}}_{i}$ denotes the health status of middle-aged and older people, and C1, C2, C3, etc., are parameters to be estimated. For the dichotomous variables, the following Probit regression model [38] was set:

(3) $${\mathrm{Pr}(Y}_{i\;}=1)=\phi {(\gamma +\beta \mathrm{internet}}_{i\;}{+\;\mu \theta }_{i\;}{+\;\varepsilon }_{i})$$

In Equation (5), i denotes middle-aged and older adults, $Y_{i}$ indicates the health of middle-aged and older adults, ${\mathrm{internet}}_{i}$ represents Internet use, $\theta_{i}$ signifies control variables included in the model, $\varepsilon_{i}$ symbolizes random disturbance terms, and β and μ are coefficients of variables of interest.

Whether or not to use the internet is a choice individuals make based on their own circumstances. There are differences in the use of the Internet by different populations. For example, younger populations are more likely to use the Internet than older populations. Therefore, the model is subject to endogeneity problems because of sample selectivity bias. This paper uses propensity-score matching (PSM) [39,40,41] to eliminate sample selectivity bias. The model divides the sample into treatment and control groups and performs a staged analysis with PSM to reduce the influence and interference of other factors. The model was set up as follows:

(4) $$Y_{i\;}{=Y}_{0i}{\;+\;(Y}_{1i}\;{-\;Y}_{0i}{)D}_{i}$$

(5) $${\mathrm{ATT}=E(Y}_{1i}{\;-\;Y}_{0i}|D_{i}=1)$$

The value of 1 in Equation (4) is the treatment variable, and when the value is $D_{i}$, individual i is in the experimental group, and when the value is 0, individual i is in the treatment group. In this study, the core independent variables were divided into two, namely, the treatment group of the middle-aged and older population who use the Internet and the control group of the middle-aged and older population who do not use the Internet. Equation (5) represents the average treatment effect for the treatment group. All analyses were conducted using STATA (version 15.0, StataCorp., College Station, TX, USA).

## 4. Results

### 4.1. Baseline Regression Results

As displayed in Table 2, Internet use demonstrated a significant effect on both self-assessed health and chronic disease status of the middle-aged and older population. Specifically, Internet use can significantly improve the self-assessed health status of the middle-aged and older population, while reducing the likelihood of chronic diseases. The results of model (A) show that the self-assessed health of Internet users will increase by 0.078 Probit units compared with non-Internet users when variables such as personal lifestyle were not included, and the model estimates drop to 0.074 Probit units when variables such as personal lifestyle were included. This result suggests that personal lifestyle is a key factor that affects the health of the middle-aged and older population. Similarly, the results of model (C) revealed that the odds of chronic disease prevalence would decrease by 0.093 Probit units for Internet users compared with non-Internet-using middle-aged and older populations when variables such as lifestyle were not included, while the model estimates decreased to 0.078 Probit units when variables such as lifestyle were included. This suggests that the effect of Internet use on the health of the middle-aged and older population may be overestimated if variables such as personal lifestyle were not controlled for. In the comparison of model (B) and model (D), Internet use had a more significant effect on improving chronic disease conditions in the middle-aged and older population than self-rated health.

Among the control variables, gender and age had a significant effect on the middle-aged and older population both in terms of self-assessed health and chronic disease status. Regarding gender, men have better self-rated health than women, and men are less likely to have chronic diseases than women. In terms of age, their health deteriorates and their chances of developing chronic diseases increase, which remain consistent with common sense. For marriage status, it was significant only for chronic disease status but not for self-assessed health. In terms of education level, it was significant only for self-assessed health and not for chronic disease status; specifically, the higher the education level of the individual, the worse his or her self-assessed health. In terms of lifestyle, people who drink tend to be much less likely to suffer from chronic diseases than those who do not alcohol. This may be related to China’s wine culture, i.e., ‘small drinks are good, big drinks hurt’. Regarding sleep duration, those who got enough sleep tended to be less likely to experience chronic diseases than those who did not get enough sleep. The results are displayed in Table 2.

### 4.2. Robustness Test

To further test the health consequences of the Internet for the middle-aged and older population, this paper uses two methods to conduct robustness tests. Since the dependent variables are multi-categorical, the Ologit model was used for the estimation of the results. Regarding Internet use, the 2018 CHARLS data also includes the question ‘Do you go online to socialize on the Internet?’ This study examines whether Internet use for socializing is the core independent variable for replacing Internet use for robustness testing, and the results of the robustness tests are exhibited in Table 3. According to the results of models (a) and (b), Internet use is significant at the 5% and 10% levels for self-assessed health and chronic disease status of the middle-aged and older population, respectively. This means that Internet use enhances the health of the middle-aged and older population as well as improves chronic disease prevalence, which is consistent with the baseline regression results. Models (c) and (d) are estimated after replacing the core independent variables. The middle-aged and older population who use the Internet for social interaction are in better health, which is consistent with the results of the above analysis. The above results indicate that the estimation results are somewhat robust.

### 4.3. Heterogeneity Analysis

The first part of this paper focuses on the effect of Internet use on the self-assessed health and chronic disease status among middle-aged and older adults and does not take into account the variability among groups. However, there are certain disparities in personal characteristics and lifestyles among the middle-aged and older population. For example, it may vary between age groups and gender groups. Therefore, we further examined the heterogeneity of the effect of Internet use on the health of the middle-aged and older population by age group, gender, and two dimensions. The results are presented in Table 4.

As presented in Table 4, there was significant heterogeneity of Internet use on self-assessed health and chronic disease status among the middle-aged and older population across sex and age groups. Regarding gender, the effect of Internet use on women’s health was more pronounced, as Internet use was effective in improving their chronic disease status and showed a suppressive effect on self-assessed health. According to the World Health Organization, those aged between 45 and 60 years are considered the middle-aged population, while those aged ≥60 years are considered older; therefore, this paper divides the age group into 45 years, 60 years, and >60 years. The results suggested that Internet use had a uniquely significant positive direct effect on both self-assessed health and chronic disease status in the middle-aged population, showing that Internet use improved the health of the middle-aged population and reduced their chronic disease prevalence. By contrast, for the 60+ age group, Internet use only had a significant effect on their chronic disease status and not on self-assessed health.

### 4.4. Endogenous Issues

The inclusion of different samples can generate sample selectivity bias because of differences in their endowment characteristics, which can bias the estimation results. To overcome endogenous problems, this study used a PSM model for in-depth analysis. Two methods are used: radius matching and kernel matching to ensure accuracy [42,43]. To ensure a good matching effect, a balance test of sample phi quality is required, no significant differences between the treatment and control groups on the main characteristic variables are noted after matching. The results of the balance test for self-assessed are presented in Table 5.

**Figure 1:**
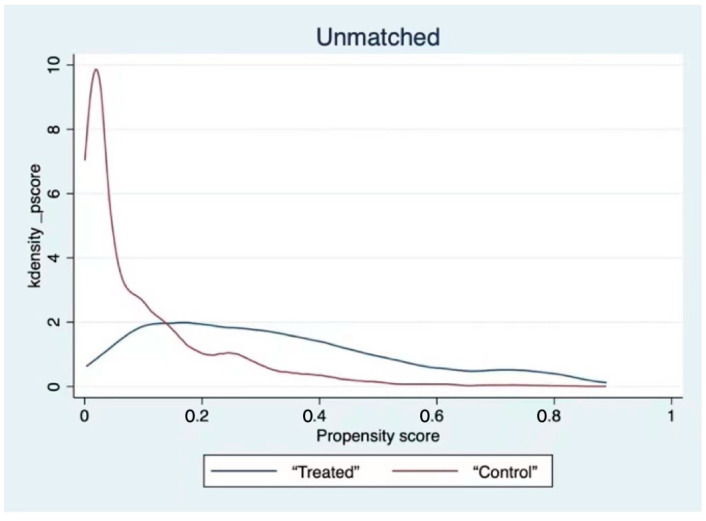
Kernel density function before matching.

**Figure 2:**
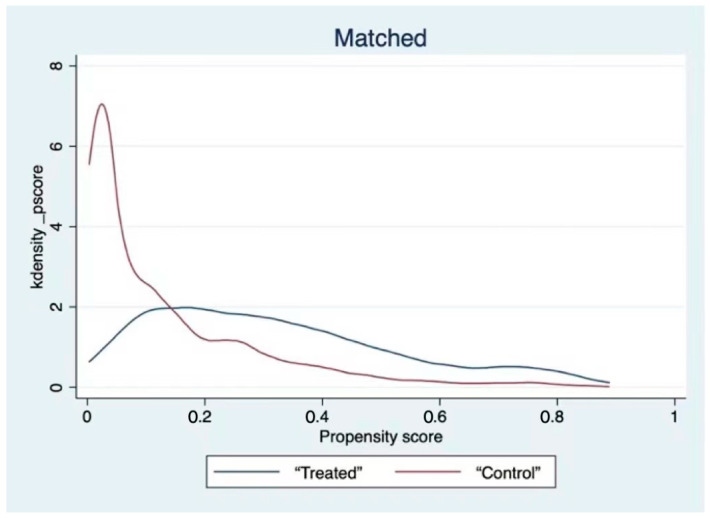
Kernel density function after matching.

As demonstrated in Table 5, the absolute values of standardized bias after matching for all variables were less than 5%. For the mean t-test, all variables passed the test. In addition, the kernel density function plots before and after matching are reported herein (Figure 1 and Figure 2). The figure shows that the curves of the matched treatment group and control group overlap to a greater extent, and the trend is more consistent. This finding revealed that the lack of systematic difference between the treatment and control groups and the matching effect is good, effectively solving the endogeneity problem caused by sample selection bias.

The average treatment effects (ATT) are reported in Table 6. According to the results, after controlling for sample selectivity bias, the health consequences of the Internet for the middle-aged and older population were 1.9% and 1.4%, respectively. The results of kernel matching are similar to these. The net effects were 2.8% and 2.0%, respectively, by nuclear matching. In all cases, the results we obtained by PSM appear robust. Thus, if the sample selectivity bias is not eliminated, the effect of Internet use on the improvement of self-rated health of the middle-aged and older population and the health consequences of the Internet on the chronic disease status of the middle-aged and older population will be underestimated.

## 5. Discussion

This paper empirically analyzed the effect of Internet use on the self-assessed health and chronic disease status of the middle-aged and older population in China using data from the 2018 CHARLS. The results revealed that Internet use had a significant effect on self-assessed health and chronic disease status in the middle-aged and older population. Compared with the middle-aged and older population who did not use the Internet, those who used the Internet rated themselves as having better health and a lower prevalence of chronic diseases, which is similar to the findings of other studies [22,24,44]. Compared with self-assessed health, Internet use had a more significant effect on the chronic disease status of the middle-aged and older population. This may be because self-assessed health is a subjective judgment based on the overall condition over time, while chronic disease status is a more objective reflection of an individual’s actual health status. The results of the heterogeneity analysis showed that the effect of Internet use on the health of middle-aged and older adults demonstrated significant heterogeneity in terms of sex and age. Regarding gender, the health consequences of the Internet for women were more pronounced. This may be because women tend to spend more time using the Internet for entertainment and socializing than men; therefore, the health consequences of the Internet are more significant. In terms of age, Internet use has a greater effect on the middle-aged population than on the older population. The potential reason is that the middle-aged population use the Internet in greater numbers, are more receptive to and understand information, and therefore make better use of the Internet to improve their health.

This study has several advantages. First, data were derived from the latest CHARLS, which can reflect the latest health status of older Chinese people and has a high representativeness and timeliness. Second, we performed robustness tests using both replacement independent variables and replacement measures to ensure the robustness of the model. Third, this study used a combination of self-assessed health and chronic disease status to measure the health of the middle-aged and older population, which can more objectively reflect a more comprehensive and objective health status of the middle-aged and older population. Fourth, we used PSM to eliminate endogeneity problems caused by sample selectivity bias and estimated the net effect of Internet use on the health of the middle-aged and older population in China.

Of course, this study also had several shortcomings. First, this study only determined whether Internet use is an independent variable to examine the effects on the health of the middle-aged and older population. However, the frequency of Internet use, duration of Internet use, and ways of using the Internet may have different effects on the health of the middle-aged and older population. This will also be the next step of our research. Second, this study only examined the effect of Internet use on the health of the middle-aged and older population using 2018 data; thus, more in-depth studies will need to be conducted using multi-year tracking data. It is important to note that this study provides general data on certain dimensions related to the health of the population but does not adequately describe each dimension. For example, in which specific dimensions does the physical and mental health of the population improve? These are all questions that we will focus on in the next step.

## 6. Conclusions

In the context of the increasing trend in aging, the comprehensive use of the Internet and digital technology to improve the health of the middle-aged and older population is one of the important ways to achieve healthy aging. Using data from the 2018 CHARLS, we examined the health consequences of the Internet for the middle-aged and older population and explored the possible heterogeneity of this effect across sex and age groups. The main findings of this paper are as follows. (1) Internet use can contribute to improving the self-assessed health and chronic disease status of the middle-aged and older population. This finding held after eliminating sample selectivity bias using PSM. (2) Some differences were noted in the impacts of Internet use on self-assessed health and chronic disease status among middle-aged and older populations. Compared with self-assessed health, the effect of Internet use on the improvement of chronic conditions among the middle-aged and older population was more pronounced. (3) The health consequences of the Internet for the population demonstrated significant heterogeneity in terms of gender and age. The impact of Internet use on the health of female and middle-aged groups was more significant than that of male and older groups aged >60 years. Finally, we found that personal lifestyle is also a key factor affecting the health of the middle-aged and elderly population. Middle-aged and older populations with healthy lifestyles tend to be healthier.

## Figures and Tables

**Table 1:** Descriptive statistics of the variables.

Variable Name	Definition	Observations	Mean	SD
Self-assessed health	Very bad = 1, bad = 2, general = 3, good = 4, very good = 5	10,778	3.684	0.753
Chronic disease status	No = 0, yes = 1	10,778	0.780	0.414
Internet use	No = 0, yes = 1	10,778	0.135	0.341
Internet use for social activities	No = 0, yes = 1	10,778	0.141	0.348
Gender	Female = 0, male = 1,	10,778	0.209	0.406
Age	unit: years	10,778	63.723	9.892
Marriage status	Married = 1, divorced = 2, widowed = 3, unmarried = 4	10,778	1.290	0.701
Education level	Illiterate = 1, primary = 2, secondary = 3, college = 4, graduate = 5	10,778	2.096	0.844
Medicare participation	No = 0, yes = 1	10,778	0.971	0.167
Smoking or not	No = 0, yes = 1	10,778	0.070	0.256
Drinking or not	No = 0, yes = 1	10,778	0.222	0.416
Sleeping time	unit: hours	10,778	6.146	2.006

**Table 2:** Baseline regression results of Internet use on health.

Variables	Model (A)	Model (B)	Model (C)	Model (D)
	Self-Assessed Health	Self-Assessed Health	Chronic Disease Status	Chronic Disease Status
Internet use	0.078 ** (0.035)	0.074 ** (0.035)	−0.093 ** (0.041)	−0.078 * (0.042)
Gender	0.141 *** (0.028)	0.117 *** (0.033)	−0.169 *** (0.035)	−0.084 ** (0.041)
Age	−0.003 ** (0.001)	−0.002 *** (0.001)	0.036 *** (0.002)	0.035 *** (0.002)
Marriage status	0.026 (0.017)	0.024 (0.017)	−0.039 * (0.023)	−0.044 * (0.023)
Education level	−0.096 *** (0.015)	−0.094 *** (0.015)	0.004 (0.019)	0.004 (0.019)
Medicare participation		−0.065 (0.065)		0.245 *** (0.081)
Smoking or not		0.067 (0.048)		−0.032 (0.060)
Drinking or not		0.014 (0.029)		−0.121 *** (0.036)
Sleeping time		−0.001 (0.005)		−0.079 *** (0.007)
Observations	10778	10778	10778	10778
Adj-R2	0.0025	0.0027	0.0582	0.0702

Note: *, ** and *** indicate significance at 10%, 5%, and 1% levels, respectively.

**Table 3:** Robustness test results of health consequences of the Internet.

Variables	Model (a)	Model (b)	Model (c)	Model (d)
	Self-Assessed Health (Ologit)	Chronic Disease Status (Logit)	Self-Assessed Health (Oprobit)	Chronic Disease Status (Probit)
Internet use	0.128 ** (0.059)	−0.117 * (0.070)		
Use Internet for social activities			0.063 * (0.035)	−0.066 (0.041)
Gender	0.182 *** (0.055)	−0.125 * (0.071)	−0.117 *** (0.033)	−0.083 ** (0.041)
Age	−0.003 (0.002)	0.063 *** (0.003)	−0.003 ** (0.001)	0.035 *** (0.002)
Marriage status	0.039 (0.028)	−0.068 * (0.041)	0.024 (0.017)	−0.044 * (0.023)
Education level	−0.162 *** (0.025)	−0.005 (0.033)	−0.094 *** (0.015)	0.003 (0.019)
Medicare participation	−0.102 (0.108)	0.425 *** (0.137)	−0.065 (0.065)	0.245 *** (0.081)
Smoking or not	0.113 (0.080)	−0.064 (0.102)	0.067 (0.048)	−0.032 (0.060)
Drinking or not	0.019 (0.048)	−0.204 *** (0.062)	0.015 (0.029)	−0.123 *** (0.036)
Sleeping time	−0.005 (0.009)	−0.136 *** (0.013)	−0.001 (0.005)	−0.079 *** (0.007)
Observations	10778	10778	10778	10778
Adj-R2	0.0027	0.0710	0.0026	0.0701

Note: *, ** and *** indicate significance at 10%, 5%, and 1% levels, respectively.

**Table 4:** Heterogeneity test results.

Variables	By Gender				By Age			
	Male		Female		45–60		≥60	
	Self-Rated Health	Chronic Disease Status	Self-Rated Health	Chronic Disease Status	Self-Rated Health	Chronic Disease Status	Self-Rated Health	Chronic Disease Status
Internet use	0.068 (0.068)	−0.024 (0.080)	−0.074 ** (0.042)	−0.110 *** (0.049)	0.074 ** (0.044)	−0.086 ** (0.049)	0.046 (0.063)	−0.154 *** (0.038)
Control Variables	Yes	Yes	Yes	Yes	Yes	Yes	Yes	Yes
Observations	2248	2248	8524	8524	4459	4459	6313	6313
Adj-R2	0.0036	0.0485	0.0028	0.0756	0.0031	0.0166	0.0045	0.0184

Note: ** and *** indicate significance at 5% and 1% levels, respectively.

**Table 5:** Sample matching quality balance test.

Variables	Unmatched Matched	Mean		Bias (%)	Reduce Bias (%)	t-Test	
		Treated	Control			t-Value	*p* > |t|
Gender	U	0.288	0.196	21.6	96.8	8.04	0.000
	M	0.288	0.291	−0.7		−0.17	0.863
Age	U	57.231	64.73	−86.5	97.7	−27.80	0.000
	M	57.239	57.414	−2.0		−0.64	0.519
Marriage status	U	1.133	1.314	−29.4	97.0	−9.19	0.000
	M	1.133	1.139	−0.9		−0.30	0.767
Education level	U	2.872	1.976	118.8	95.8	40.35	0.000
	M	2.871	2.833	5.0		1.40	0.163
Medicare	U	0.983	0.969	9.4	83.1	3.01	0.003
	M	0.983	0.981	1.6		0.49	0.625
Smoking or not	U	0.106	0.065	14.6	93.2	5.65	0.000
	M	0.105	0.103	1.0		0.24	0.808
Drinking or not	U	0.407	0.194	47.8	94.5	18.43	0.000
	M	0.406	0.395	2.6		0.64	0.520
Sleeping time	U	6.308	6.121	10.4	88.0	3.32	0.001
	M	6.309	6.331	−1.2		−0.37	0.709

**Table 6:** Results of PSM estimation.

	Self-Assessed Health				Chronic Disease Status			
	Treated	Control	ATT	SE	Treated	Control	ATT	SE
Unmatched	3.699	3.681	0.017	0.021	0.688	0.794	−0.107	0.011
Matched								
Radius neighbor matching	3.696	3.678	0.019	0.026	0.688	0.702	−0.014	0.015
Kernel matching	3.698	3.670	0.028	0.026	0.687	0.707	−0.020	0.015

Note: The radius of radius neighbor matching is 0.01, and default values are used for both kernel function and bandwidth in kernel matching.

## Data Availability

The data of 2018 China Health and Retirement Longitudinal Study (CHARLS) is publicly available at http://charls.pku.edu.cn/pages/data/2018-charls-wave4/zh-cn.html accessed on 24 September 2020.
